# Modern Therapeutics and Pharmacy*Read at San Antonio meeting Fifth District Medical Society, February 8, 1905.

**Published:** 1905-03

**Authors:** Frederick Hadra

**Affiliations:** San Antonio, Texas


### TEXAS MEDICAL JOURNAL.

ESTABLISHED JULY, 1885.

PUBLISHED MONTHLY.—SUBSCRIPTION $1.00 A YEAR.

VOL. XX. AUSTIN, MARCH, 1905. No. 9.

### Original Contributions.

For Texas Medical Journal.

### Modern Therapeutics and Pharmacy.*

FREDERICK HADRA, M. D., SAN ANTONIO, TEXAS.

The title of my paper will cause you to anticipate much more than you will receive, and its disorderly presentation will justify legitimate criticism; but the subject is so complex that I must ask you to overlook its awkward preparation. I also wish to disclaim any personal feeling toward the committee on proprietary drugs, whose recent circular letter and list of questions induced me to select my subject. Whenever I refer to the committee it should be understood that I refer only to its principles and not to the gentlemen composing it.

If our society were merely an obscure country organization, then any literature promulgated by its authorized agents, particularly if it partook of the nature of extreme radicalism, could be passed over as of little moment; but when such literature emanates from a society as representative of progress as ours is, or should be, including, as it does, in its ranks officers and members of national and State societies, then I deem it justifiable to enter a protest against our committee's indiscriminate attack upon everything smacking of advancement in a therapeutical and pharmaceutical di-

*Read at San Antonio meeting Fifth District Medical Society, February 8, 1905.

2—Mj rection, and I sincerely hope that the great majority of this society will agree with me.

At least 75 per cent of all people seeking medical advice and treatment can be relieved by natural therapeutics alone. Natural therapeutics, as defined by Potter, "including the operations of the Vis Medriatrix naturae,—the healing power of nature,—those modes and processes of healing which occur independently of art, and tend to spontaneous decline and cure of disease." Fresh air, diet, exercise, and an occasional cleaning out, will still further reduce the percentage of those requiring the aid of *applied therapeutics;* or the application by art of agents foreign to the living organism, for the purpose of aiding nature to restore the body to a healthy condition.

It is this natural law which is partly responsible for that ever-increasing horde of irregular healers met with everywhere. I say only *partly* responsible advisedly, for we ourselves are mostly to blame for that large stampede of patients to the camp of the enemy, the homeopaths, osteopaths, Christian scientists, etc. If when this large number of imaginary or but slightly ailing class of sufferers come to us for treatment, we could, like our more wise competitors, give them doses appealing to both eye and palate, instead of powders, pills, and liquids, the very sight or smell of which, let alone their taste, would nauseate a wooden Indian, then I say we might regain our former prestige and gather back into our fold that large class of timid sufferers who are now afraid to come to us even when *really* in need of skillful medical attention and therapeutics.

If reforms are needed in our therapeutics, whether it be in the too frequent use of so-called patented or proprietary remedies, or what not, calm and conservative discussion and appeal to reason will accomplish much more good and bring about a much more rapid reform than fanatical attacks and indiscriminate condemnation of a whole system. Over-zealousness has always defeated or delayed the accomplishment of every reformation however urgent its necessity. This rule is particularly applicable to the subject under discussion. No one will dispute that the great and ever-increasing numbers of proprietary and patented remedies annually presented to and urged upon physicians through agents, circulars, and the medical press, are confusing enough to drive a man to drink, in an effort to cull from this heterogenous mass that which is really deserving of adoption; but it also can not be disputed that it is a necessary part of our duty to undertake the task of sorting out, and no progressive merchant or mechanic worthy of the name is exempt from a similar duty toward his patrons. Is it not a confession of weakness on our part to cry out in great alarm that the manufacturing chemist is usurping our domain and is forcing us to use his wares against our important wills?

Who is responsible for the manufacture of these numerous preparations against which the committee protests so fearfully? Do you suppose that Messrs. Merck, Stearns, Wyeth, Mulford, Sharp & Dohme, Park Davis, Fairchild, etc., would expend hundreds of thousands of dollars annually and employ scores of able chemists and pharmacists in their laboratories for research if there were no appreciation and demand for the innovations constantly made to our medical armamentarium, as the result of their labor?

Not only in our own profession, but in all the other arts and sciences, men are bending every energy toward advancement and perfection. Only the other day I read of a certain United States Senator who lost his seat in Congress once because he voted in favor of expending a few thousand dollars to enable the inventors of telegraphy to conduct their first experiments. To this day there are thousands of people, and among them doctors, who are opposed to vaccination, vivisection, diphtheria antitoxin, etc., and yet the world moves on and these mossbacks are forced aside to make way for the onward procession of progress.

A pharmacopœia is an official list of the drugs and their preparations recognized by the medical profession of a certain country. In the United States its publication is left to the medical and pharmaceutical profession and it is revised every ten years by a convention called for that purpose. If, therefore, we would confine ourselves strictly to official drugs and remedies, it might be conceivable that at times we might have to wait from five to ten years before adopting such universally recognized therapeutic agents as the different antitoxins and serums, notably diphtheria, glanders, tetanus, erysipelas; the glandular extracts, like adrenaline, thyroid, pituitary, pancreatin, etc.

Local agents, like the X-ray, Finsen light, etc., and such useful, we might say, almost indispensible daily agents, like the *antipyretics,* phenacetine, antipyrine, phenocol hydrochlorate, and exalgin.

Silver salts, like actol, argentamin, picratol, protargol, largin, itrol, and collargol.

Antiseptics, like icthyol, formalin, aristol, iodol, benzozol, xeroform, lysol, trikesol, hydrozone, creoline, and nosophin.

Intestinal and pulmonary antiseptics and astringents, like benzol, alphozone, orphol, dermatol, tannigen, tanalbin, guiacol, duatol, benzosol, protan, acetozone, creosotal.

Local and general anesthetics, like cocaine B., orthoform, nirvanine, pental, etc.

Hypnotics, like veronal, chloralmid, hypnal, sulphonal, trional, somnose, urethan.

Genito-urinary antiseptics, diuretics, and uric acid solvents, like helmitol, formin, urotropin, cystogin, diuretin, piperazin, lycidin, lycetol, methyelene blue, etc.

Antirheumatics, like salipyrin, salophen, salicen, and malakin.

Of so-called proprietary and copyrighted preparations I will mention only a few of those which have become almost every-day adjuncts in our daily practice:

Of the digestants, elixir lactopeptine, essence of pepsin, extract of pancreatin, peptogenic milk powder, liquor diastas, etc.

Of foods, Mellins', Horlick's, malted milk, panopeptone, bovinine, bovril, liquid peptonoids, malt extracts, etc.

Of antiseptics, glycozone, glyco-thymoline, euthymol, borolyptol, phenal-sodique.

Of tonics and alteratives, pepto-mangan, arsenauro, mercauro, glycerophosphates, emulsions of codliver oil, etc.

Of laxatives, cascara compounds, tamarinds comp., so-called fruit salts, and effervescent salts of various kinds.

And lastly, such preparations as apiol, aconitina, aneasthesin, sea sickness, bromoform, camphoric acid, chloretone, digitalin, um verum, digitalone, for hypodermic, 5-10 minims; duboisine, mydriatic, like belladonna, less irritating; ergotol, ergotin, gonosan, kava-kava in santal oil; hydragogin, napelline, aconite, oil of origeron, physostigmine salicylate, salicylos, stypticin, catarmine hydrochlor ulerin styptic.

The morphine derivatives and preparations, chloranodyne, heroine, dionin.

The iodine preparations, iodophin, aristol, europhen, iodol, nosophen.

The iron preparations, ferratin, and other albumates, arsenates, and bromates.

The guiacal preparations, guiacol, carbonate or duotal, creasote carbonate or creosotal, and benzosol, 54 per cent.

The tasteless quinine preparations, febriline, cocoquinine, Kairin, thalin, hydroquinine, euquinine.

Sulphur derivatives, thiginal, thiocal, ichthyol, thiol, ichthalbin.

Bromine derivatives, bromoform, bromipin.

None of the long list picked out at random are official; many of them are synthetical preparations and of the compounds hardly a one mentioned is not of some use.

I should be sorry, indeed, if the prejudices of any member of this society should so far overcome his better judgment as to banish all or most of these drugs from his practice without investigating their merits. So also if we desire a local antiphlogistic effect, and have to choose between the ancient, unsightly, unhygienic and troublesome flaxseed poultice and the newer proprietary article called antiphlogistine or its numerous imitations, a physician must needs be prejudiced, indeed, who will prefer the former. I should also feel sorry for the patient whose physician would insist upon taking the acid tincture of chloride of iron for long periods when such elegant and well-established preparations as Gude's Pepto-Mangan and other ferraginous tonics are at his disposal.

We are also under obligations to the manufacturing chemists for such elegant gynecological adjuncts as the antiseptic vaginal tampons, the elastic vaginal and urethral suppositories, the perfect aseptic antitoxin syringes, etc.

It would certainly be injudicious, to say the least, for any physician to eliminate from his practice most or all of the various preparations, simple and compound, simply because some of them were patented or the names of some of the mixtures or combinations copyrighted or registered.

A correct diagnosis must, of course, precede every successful therapeutic attack, but once having made the diagnosis much of the subsequent success or failure may depend upon the form of medication we select, as every practitioner has often learned to his sorrow. Having, for instance, made the diagnosis of a certain form of anemia in which arsenic is demanded, it may be a matter of theoretical indifference which preparation we prescribe, but it may be quite a different matter with the patient, who has to take it for long periods. What does he care how much intelligence has been displayed in his diagnosis, and how great is his physician's knowledge of materia medica, if his stomach and senses rebel against the drugs that are being forced upon him? He wants to get relief, and, therefore, whatever remedy is selected must be given in such form that he will take it, and his stomach retain it. Otherwise neither the correct diagnosis nor the correct selection of the drug will have any practical result whatever. It is certainly the height of folly to insist upon a patient taking an obnoxious preparation simply because our pharmacopia so directs, when a more elegant and palatable one may be gotten from our manufacturing chemist who has, perhaps, devoted years of labor toward attaining perfection in that particular preparation. Elegance and palatability are especially desirable for that large class of neuresthenics and hypochondriacs, so frequent among women and children, who are now driven to more attractive healers who make up by shrewdness what they lack in scientific attainment. If we should pay more attention to these minor details of therapeutics and, instead of attacking those who would help us, encourage them in their effort, there would be less need for outside legislation to suppress our more wily and unscrupulous competitors.

Does it not strike you as somewhat incongruous that we alone of all professions and trades should rise up in arms against a coordinate branch which is continually striving to assist us in improving our therapeutic weapons? Suppose railroad, steamship, telephone, and electric light companies, etc., should suddenly promulgate orders to the effect that henceforth all innovations in life-saving, time-saving, etc., must be stopped, because inventors are forcing upon them improvements so fast that they can not cull the useful from the useless. Our medical societies would be among the first to enter a protest, and yet our committee is urging us to do this very thing. If, instead of condemning the whole system of medical sample business, we would take advantage of the opportunities offered to make intelligent selection only of such preparations of drugs of reliable concerns as appeal to reason and common sense, those of us who do so will certainly have an advantage over those who do not.

Conservatism in therapeutics, as well as in every other branch of science and government, is commendable up to a certain point; after that point is passed it is no longer conservatism, but mossbackism. We style ourselves regulars, scorn the sobriquet allopath, and resolve and resolute at every meeting against pathics of all kinds. Our chief claim for superiority over all other so-called schools has always been that we accept innovations from any and every source tending to the advancement of our profession regardless of its origin—as we did vaccination from a milk man.

Even the medical department of the army, one of the formulators of our official pharmacopœia, and most conservative in all things therapeutic, approves of no less than *seventeen* unofficial preparations, several of which are patented or registered as proprietary, as indicated by the following list taken from its latest supply-table: Adrenalin hydrochlorate, albolene, antipyrine, antitoxin diphtheria, digitalinum, ergotinum, glycozone, hydrozone, ichthyol, peptonizing tablets, phenacetine, salophen, sulphonal, trional, urotropin, trichisol, and malted milk.

Now for the circular itself: The committee assures us that "great damage is done the people (and the physician) by the medical sample business, along with the prescribing druggist." I had always thought that the medical sample business was practiced exclusively with physicians, and it never occurred to me before that the dear people were damaged by Mr. Jones' agent leaving a new preparation of cod liver oil, cascara compound, pepsin compound, cough mixture, etc., upon my desk; neither had I discovered before that I was being damaged by these Chadwickean representatives of the manufacturing chemists. I had always imagined that I had judgment and intelligence enough to discriminate, without the agent's advice, which of the preparations presented deserved consideration and which did not, and notwithstanding the great danger I am assured of, I hope that the medical sample business will continue as long as I am capable of practicing my profession. When my mind becomes so feeble that irresponsible agents can dictate to me what remedy I must use, then I will retire and give way to men who have minds of their own.

I must be very dense, indeed, for I can not see what connection the medical sample business has with the prescribing druggist. As long as I can remember druggists have been in the habit of prescribing for all sorts of diseases. Before the more convenient modern preparations were obtainable, such crude drugs as quinine, calomel, castor oil, Epsom salts, santal middy, copaiba, paregoric, flaxseed meal, all sorts of herbs, turpentine, carbolic acid, etc., were prescribed by him. Would our committee insist that because of this fact all the above and many more should be banished from our routine practice?

The committee further says, "the fact can not be controverted that three-fourths of the prescriptions received by pharmacies are for proprietary remedies advertised to doctors only. The druggist simply opens the package and writes: "Teaspoonful three times daily, after meals." The original bottle is given the patient, and he does not go back to see the doctor, etc., but goes back and asks

"for more of the same." I must again confess my inability to see any point in this argument against the use of proprietary remedies, for if the druggist will grant the request of his customer to have any medicine repeated without an order from the original prescriber he will, if he is paid for it, repeat an extemporaneous prescription just as readily as a proprietary one, and if the doctor was intelligent in the selection of his first proprietary prescription, there should be no more harm to the patient if he has it refilled, than if it had originally been an extemporaneous one. The evil complained of lies not with the form of the remedy prescribed, but with the cupidity of the druggist who prostitutes his calling and infringes on our rights. An excellent example of this was furnished during the great dengue epidemic here in 1897. When the disease first made its appearance I had many calls. I did not prescribe a proprietary remedy in any case, yet as the epidemic progressed and as calls should have increased, they grew less. The explanation was easy. If you were called to the family of Mr. A., living on X street, say you left a prescription for the husband. It relieved him, and next day when Mrs. A. was taken ill, she had the prescription repeated for herself without calling a doctor. Mrs. B., living next door saw how nicely her neighbor got along and, without sending for a physician, had the same prescription refilled for herself and children; and so, one or two calls on Mr. A. relieved many families of the necessity and expense of sending for a doctor at all. If I am called to treat a sprain of the ankle, and find it necessary to order an antiphlogistic application, it would be just as easy for the patient to send to his druggist daily for more flaxseed meal or iodine, as it would be for him to order more cans of the more cleanly proprietary preparation antiphlogistine. A tonic or cough medicine, or quinine mixture or capsule, would share the same fate, whether proprietary or extemporaneous. Returning to the circular, it there quotes a writer, said to be a manufacturer, as follows: "For one physician capable of prescribing the precise medical agent needed by an individual patient there are at least five who prescribe proprietaries. They are the chief stand-by of the country practitioner." "If these statements are true," says the committee, "it would appear that the standard of education among medical men in this country is very low, indeed." Here, again, I differ from the committee's deductions. Even if the proportion of proprietaries given was correct, which I doubt, it would not necessarily follow that our standard of education is very low, for, as I stated before, the diagnosis in every case is the essential and most difficult matter to be determined, and no uneducated person, to whatever school he may belong, can make many correct diagnoses if he is as uneducated as our committee would have us believe. Having once diagnosed the case, there seldom exists, except in the minds of theorists, the necessity for such wonderful therapeutic preciseness that each individual patient must have an entirely different medical combination quantitively and qualitatively. If I have ten different patients all having approximately the same pathological condition of their lungs, and I think heroin, for instance, indicated, I fail to see why glycoheroin should not be used for all ten whether they are all sick at one time in one hospital or are all scattered over different parts of town and are taken sick at different times. This principle is adopted in all large hospitals the world over, where favorite prescriptions of famous professors are often prescribed entirely by number or abbreviation for cases of similar nature. Potter hits the nail on the head when he says, "It is worse than futile to attempt to prescribe for every symptom. The underlying cause of the bodily condition must be sought out and prescribed for simply."

I believe we have but few important drugs today that can not be prescribed without detriment to the patient in some more attractive form or vehicle than can be extemporaneously prepared at an average drug store. The country doctor especially has cause to thank the manufacturing chemist for putting at his immediate disposal every drug in our materia medica, simple and in combination, in such form that he can rely implicitly upon both the quantity and quality of every dose he wishes to give.

Would our committee have him discard his tablet triturates, compressed tablets, pills and accurately prepared mixtures for the old-time way of measuring out on the end of his barlow dosages for men, women, and children; or trusting the preparation of his liquids, ointments, and pills to some out-of-the-way drug store with an underpaid clerk, an antique stock of moth-eaten roots and herbs, or stale precipitated tinctures and old extracts of uncertain strength, weighed on scales of doubtful accuracy, measured out and mixed in graduates, mortars and pill tiles reeking perhaps with past offenses, and finally put up in bottles rinsed out in water of slop-like purity? If any member present thinks that I have overdrawn this picture, let him investigate for himself, and then tell me that he would not rather take a tablet or mixture prepared by some of our standard manufacturing chemists than to trust his own dose to any and every shop that bears the sign of pharmacist. Why, the committee itself makes a powerful argument in favor of original packages when it calls our attention to the fact that "some druggists have even a favorite remedy of their own make, which they prescribe or substitute when filling a prescription from a regular physician." It is said that a certain "board of health, suspecting that prescriptions for phenacetine were being filled by druggists with acetanilid, or a mixture of phenacetine and acetanilid, sent inspectors to obtain definite information on the matter. Altogether 373 samples of phenacetine were brought from as many different drug stores, phenacetine being especially called for, and in some instances, obtained on physician's prescriptions. Of the 373 samples, 58 were pure phenacetine, 315 were adulterated with cheaper drugs, mainly acetanilid, while 32 samples were pure acetanilid. What do you think of this for an argument *against* proprietary remedies? Do you wonder that reliable manufacturing chemists with a reputation at stake, should urge us to prescribe original packages whenever it is possible to do so?

From the past paragraph one would infer that the committee had awakened from its dream to the real danger confronting us; but,

"Alas! from what high hope to what relapse, unlooked for are we fallen!"

Listen to this pitiful appeal: "The patent medicine men are wide awake, etc. Shall we remain idle until we are bound neck and heel? Is it fair to our patients? Do not our obligations to humanity demand that, as scientific and honest men, we openly declare our opposition to this nefarious business?" i. e., the manufacturer of patent, proprietary and synthetecal medicines. In marked opposition to the views held by our committee on the harmful and illegal traffic caused by synthetecal drugs is that of Torald Sollman, who, in a scholarly discussion on "Pharmacology," in the *Journal of the American Medical Association*, of November 25, 1903, says, regarding the study of vegetable drugs, which as yet are so little understood, "that it does not seem too much to hope that we may eventually be able to dispense entirely with vegetable drugs and produce artificially the identical constituents which we now use and in many cases improvements on these."

I will conclude my paper by briefly answering the list of questions accompanying the circular.

First question: "Do you personally approve the use of patent and proprietary drugs in a physician's practice?" I do not believe that the committee had in mind Peruna, Mrs. Lydia E. Pinkham's and other life saving preparations, when it used the term patent. Unless it did I answer the question affirmatively. I will go even further, and say that I would consider it a reflection on the progressiveness of the personnel of this society if any member thereof should unqualifiedly answer it negatively.

Second question: "Have you analyzed said drugs? And if so, did you find analysis to correspond with the formula submitted by the manufacturer?" I will answer this question by asking the committee another equally as pertinent: "Did you ever analyze your hypodermic strychnine, morphine, nitroglycerine, and atropia tablets, upon which you rely to save life in great emergencies?" If you have not so analyzed them quantitatively and qualitively, why are you not criminally negligent in not so doing, if you suspect the same manufacturer who prepares these tablets and the alleged unreliable nostrums, which you decry, of deceit and misrepresentation? Can you afford to unhesitatingly employ one of their products which require the greatest possible amount of care, skill and honesty, and yet suspect them of willful misrepresentation in another product? Again, if these manufacturers can not be trusted in placing before you certain palatable and elegant-looking tonics, cough mixtures, antiseptics, etc., why do you trust these same parties in supplying you with the fluid extracts, tinctures, etc., used in your extemporaneous prescriptions? Is it the custom of the members of this committee to analyze every bottle of fluid extract, tincture, powder, etc., before they permit them to go into their prescriptions?

But of all the questions, the third is the most startling of all:

Third question: "What per cent of the present great increase in insanity do you attribute to the use of these nostrums?"

I am not an expert on insanity, nor have I ever devoted special study to the subject; but unless some wonderful changes have occurred in the realm of psychiatry, I must confess total ignorance of any etiological connection between the various preparations of cascara sagrada, iron preparations, Gude's Pepto-Mangan, the hypophosphites, cod liver oil, antiphlogistine, baby foods and insanity.

And to the fourth question: "What per cent of the increase of inebriates and dope fiends do you attribute to their use?"

I can not recall a single dope fiend or inebriate during my practice of seventeen years whose affliction I could trace, by the most devious and hypercritical analysis, to proprietary drugs. Perhaps the committee, though, has been more keen in this respect, or has up its sleeve certain preparations of which I have never heard, for I am positive that I have never myself used any remedy which could even remotely have been held responsible for such disastrous results. Furthermore, I firmly believe that if dope fiends, particularly cocaine fiends, are created by doctors at all, at least 95 per cent are so made by throat and nose specialists, in the necessary treatment of most of these diseases, and not by the general practitioner, who seldom uses this drug more than once or twice on the same patient. A more pertinent question would have been: Do you personally approve the hypodermic use of morphine in a physician's practice? Then questions three, four, five, six, and seven might more logically have followed, for no one can deny the baneful effect of the too ready and frequent use of the hypodermic syringe on susceptible individuals in causing dope fiends. And yet, I am quite sure that no member of the committee would suggest that the hypodermic use of morphia should be eliminated from the physician's practice.

Another stunner is question number five: "What influence have they on the unborn child?" I should say that they, the better, newer drugs and proprietary preparations, should have a very beneficial effect on the unborn child if taken either by the father or mother. For instance, a course of arsenauro, mercauro, Gude's Pepto-Mangan, etc., would greatly benefit an anemic mother, and some of the phospho-lecithin compounds, or locally, some of the newer silver preparations, might help the father considerably, and save the offspring an attack of ophthalmia neonatorium.

Sixth question: "Shall we protect the laity against their use? And how?"

If the intelligent use of the drugs mentioned is not injurious per se, why should we protect the laity against their use any more than against the employment of any other drugs? Would the committee advocate the abandonment of calomel, castor oil, magnesium sulphate, quinine, flaxseed meal, paregoric, laudanum, carbolic acid, etc., because the laity can also go to the drug store and purchase these just as they can cascara preparations, phenacetine, euthymol, listerine, etc.?

Seventh question. "Shall we protect the profession while we may, or shall we wait until the majority are on the other side?"

As I have never before considered our profession in danger from any of the causes mentioned, I must confess that I prefer to wait until the majority are on the other side; perhaps the pressure will then be relieved somewhat on this side, and many of the anticipated dangers will right themselves.

